# *Trypanosoma evansi*: Molecular Characterization and Validation of a Cathepsin-L-like Peptidase as a Potential Therapeutic Target

**DOI:** 10.3390/pathogens15090954

**Published:** 2026-09-08

**Authors:** Adolfo Bremo, Yenis G. Pérez, Lilia M. Turcios, Conor R. Caffrey, Matthew Bogyo, James H. McKerrow, Marisa Gonzatti

**Affiliations:** 1 Grupo de Bioquímica e Inmunología de Hemoparásitos, Departamento de Biología Celular, Universidad Simón Bolívar, Sartenejas 1080, Estado Miranda, Venezuela; adolfobremo@gmail.com (A.B.); yenisperez@usb.ve (Y.G.P.); liliaturcios@uky.edu (L.M.T.); 2 Laboratorio de Bioquímica, Programa de Ciencias Veterinarias, Universidad Nacional Experimental Francisco de Miranda, Coro 4101, Estado Falcón, Venezuela; 3 Tropical Disease Research Unit, Department of Pathology, University of California San Francisco, VAMC, San Francisco, CA 94121, USA; ccaffrey@health.ucsd.edu (C.R.C.); jmckerrow@health.ucsd.edu (J.H.M.); 4 Pathology Department, Stanford University, Stanford, CA 94305, USA; mbogyo@stanford.edu

**Keywords:** *Trypanosoma evansi*, cysteine peptidase, K11777 (SLV213), drug target

## Abstract

C1A cysteine peptidases (CPs) are major contributors to host–parasite crosstalk and have been proposed as anti-trypanosomal chemotherapeutic targets. *Trypanosoma evansi* is the most widespread animal trypanosome of African origin causing the trypanosomosis known as “Surra” or “derrengadera”. Gelatin zymography combined with peptidase class-specific inhibitors identified four CPs in *T. evansi* lysates with relative masses of 28, 31, 36 and 40 kDa (p28, p31, p36 and p40). The CPs present in the *T. evansi* lysate preferentially hydrolyzed the Z-Phe-Arg-AMC fluorogenic substrate over Z-Gly-Arg-AMC and Z-Ala-Arg-AMC, and the hydrolysis of Z-Phe-Arg-AMC was inhibited by the K11777 (also known as SLV213) vinyl sulfone inhibitor with an apparent IC_50_ of 3.06 nM. Active-site labeling using the ^125^I-LHVS-PhOH vinyl sulfone inhibitor confirmed the identity of p28, p31, p36 and p40 as cathepsin-like peptidases. Partial purification of the *T. evansi* p40, analysis of its N-terminal peptide sequence and cross-reactivity with an anti-*T. brucei* (*Tbr*)CATL antiserum revealed its orthology to the C1A CPs present in *T. b. brucei*, *T. cruzi*, *T. dionisii* and *T. rangeli*. Treatment of infected mice with the pan-cathepsin irreversible inhibitor, K11777 (10 mg/day), for 14 (*n* = 5) or 17 (*n* = 3) consecutive days was trypanocidal and significantly increased mice survival. These results validate *Tev*CATL as a potential therapeutic target for the treatment of animal trypanosomosis caused by *T. evansi*.

## 1. Introduction

*Trypanosoma evansi* is an African kinetoplastid pathogen within the Trypanozoon subgenus that causes the trypanosomosis known as “Surra” in the Old World and “mal de caderas”, “derrengadera” or “murrina” in Latin America [1,2]. In Northern Africa, the Middle East and Asia, the disease affects domesticated and wild mammals, mainly camelids, equids and dogs, while in Central and South America the main reservoirs are equids, buffaloes, cattle, dogs, capybaras and other wildlife [2,3,4,5]. Imported animals with *T. evansi* infections have been reported in Europe [6,7]. In South America, the parasite is mechanically transmitted by biting flies (tabanids and *Stomoxys* spp.), by vampire bats, *Desmodus rotundus,* as a biological vector, and by iatrogenic procedures [2,3,4,5]. The first documented case of human *T. evansi* infection was reported in an immunocompromised Indian patient who lacked the trypanolytic factor associated with ApoL1 [8,9]. *T. evansi* infection was also described in an individual in Vietnam with no genetic Apo-L1 deficiency [10].

The Trypanozoon subgenus also includes *T. brucei brucei*, which causes “nagana” in cattle, *T. b. rhodesiense* and *T. b. gambiense* which cause Human African trypanosomiasis (HAT or sleeping sickness) and *Trypanosoma equiperdum,* the etiological agent of “Dourine” that affects equids [1,4,11]. Genetic studies support the reclassification of *T. evansi* and *T. equiperdum* as subspecies of *T. brucei*, i.e., *T. b. evansi* and *T. b. equiperdum,* since they evolved in multiple independent events from genetically distinct *T. brucei* strains and share a high degree of genomic similarity despite significant biological differences [12,13,14]. Phylogenetic and clustering analysis based on 168 single-copy orthologous genes demonstrated a close relationship between *T. b. gambiense* DAL972 and two *T. evansi* strains, STIB805 and YNB [13,15], which segregate from the *T. b. brucei* TREU 927/4 [16] and *T.b. equiperdum* OVI clades [14]. Whole-genomic studies showed the emergence of four different clades of related pathogenic trypanosomes, with distinct evolutionary origins: *T*. *b. evansi* type A and type B evolved from *T. brucei* in Western and Central Africa, whereas *T. b. equiperdum* type OVI and type BoTat evolved from *T. brucei* in Eastern Africa [14,17]. Lukeš et al. (2022) [18] proposed that the Trypanozoon (sub)species, resulting from a monophyletic origin, be designated as *T. brucei* ecotypes resulting from independent evolutionary paths (polyphyly).

Kinetoplastid peptidases participate in parasite nutrition, lysosomal protein turnover, host cell invasion and signaling, and are essential for infectivity and survival in the host [19,20,21,22]. In addition, the immunogenicity of trypanosomatid peptidases has encouraged their use as diagnostic tools and experimental vaccines [23,24,25]. Cysteine peptidases (CPs) of the Clan CA, family C1 [26] degrade host immunoglobulins and promote the turnover of the variant surface glycoproteins (VSGs) that cover the surface of *T. brucei* [19,27]. CPs are also major factors in the pathogenesis of HAT, one of the most important neglected tropical diseases (NTDs) affecting humans in sub-Saharan Africa [19,20,21]. The *T. brucei* rhodesiense cathepsin L-related CP (*Tbr*CATL) [28,29] facilitates parasite entry into the central nervous system, an essential step for the neurological stage of the disease [30,31], while the *Tb*CATL has been implicated in extravasation in endothelial cells, which contributes to cardiac dysfunction [32].

Chemical validation using CP inhibitors has demonstrated that the *Tbr*CATL lysosomal peptidase is essential for parasite survival and is therefore a potential drug target [33,34]. Several CP inhibitors, including synthetic vinyl sulfones that engage *Tbr*CATL are effective in vitro and in vivo against *T. brucei* [29,35,36,37,38]. This includes the CP inhibitor, K11777, or SLV213 (N-methyl-piperazine-Phe-homoPhe-vinyl sulfone-phenyl), which has been validated as a broad-spectrum anti-trypanosomal agent [20,29,37,38,39,40,41,42,43].

Here, we characterize the major *Tev*CATL peptidases and demonstrate that their inhibition by K11777 kills *T. evansi* and extends host survival in a mouse infection model.

## 2. Materials and Methods

### 2.1. Parasites

The *T. evansi* capybara strain from Venezuela (TeAp-ElFrío01) was expanded in Sprague-Dawley rats as previously described [44,45]. Parasites were purified by anion-exchange chromatography using DEAE-cellulose [46], quantitated in a Neubauer chamber and stored in liquid nitrogen until use.

### 2.2. Peptidase Identification and Characterization

Purified trypanosomes (6 × 10^8^ parasites) were resuspended in 1 mL Tris saline hypotonic buffer (TSH; Tris-HCl 10 mM, pH 7.4, NaCl 20 mM) and lysed with three cycles of freezing and thawing. The extract was clarified by centrifugation at 5000× *g* for 30 s and the protein concentration determined [47]. The soluble lysate (30 μg/lane) was analyzed by 0.1% gelatin zymography using 12.5% polyacrylamide gels [48]. Zymograms were washed three times each for 5 min with distilled water at room temperature (RT), once in 100 mM Tris-HCl, 1%Triton X-100, pH 8.8, for 30 min at RT and then incubated in 100 mM Tris-HCl, 2.5 mM CaCl_2_, pH 7.2, with or without 25 mM cysteine for 30 min at 37 °C. Peptidase activity was identified as clear bands after staining with 3% Coomassie Brilliant Blue R-250 in 40% methanol, 10% acetic acid, and then destaining in the same solution minus the Coomassie Blue [48].

The pH profile of *T. evansi* peptidases was analyzed by 0.1% gelatin zymography of the trypanosome lysate (20 μg/lane). The resolved peptidase activity was activated at 37 °C for 30 min using the following buffers supplemented with 25 mM cysteine, 100 mM sodium citrate (pH 3.0–6.0), 100 mM Tris-HCl (pH 7.0–8.0) or 100 mM sodium bicarbonate (pH 9.0–10.0).

Susceptibility to various peptidase class-specific inhibitors (Sigma-Aldrich, St. Louis, MO, USA) was tested by 0.1% gelatin zymography of parasite lysates (20 μg/lane). Zymograms were incubated in 100 mM sodium citrate buffer, pH 4.0, with 25 mM cysteine at 37 °C for 30 min in the presence of one of the following inhibitors: 1 mM PMSF that inhibits serine peptidases, 10 mM EDTA (a metallo-peptidase inhibitor), 1 µM pepstatin (an aspartic peptidase inhibitor), 1 mM iodoacetic acid (IAA—a CP inhibitor), 20 µM leupeptin (inhibits serine peptidases and CPs) or 10 µM E-64 (a CP inhibitor). Control assays were incubated under the same conditions with no inhibitor or with 10% DMSO.

Four acrylamide-conjugated equine proteins: hemoglobin, fibrinogen, albumin and immunoglobulin A (IgA) (Sigma-Aldrich, St. Louis, MO, USA) were tested as substrates for the *T. evansi* lysates (20 μg/lane) using 0.03% zymography [49,50]. Degradation of hemoglobin, fibrinogen, albumin and IgA was examined at pH 7.0, to mimic the pH of the cytosolic and extracellular compartments. Activation of all zymograms was carried out in 100 mM Tris-HCl buffer, pH 7.0, supplemented with 25 mM cysteine and 2.5 mM CaCl_2_, at 37 °C for 30 min. Hemoglobin degradation was also assessed by incubation in 100 mM sodium citrate buffer, pH 5.0, supplemented with 25 mM cysteine and 2.5 mM CaCl_2_, at 37 °C for 30 min to simulate the pH of the lysosomal compartment.

### 2.3. Measuring T. evansi CP Activity with Fluorogenic Peptidyl Substrates

The trypanosome lysate (2 × 10^6^ parasites/mL) was tested with three fluorogenic dipeptidyl substrates (4 µM): Z(benzyloxycarbonyl)-Phe-Arg-AMC (7-amido-4-methyl-coumarin), Z-Arg-Arg-AMC and Z-Gly-Arg-AMC (Sigma-Aldrich, St. Louis, MO, USA), as described in [51] with minor modifications. The assay contained 100 mM sodium phosphate buffer, pH 7.0, 10% DMSO and 2 mM DTT in a final volume of 2 mL and was incubated at 30 °C for 10 min. Fluorescence was measured every minute using a Shimadzu Plus fluorimeter (RP-5301 PC) (Schimadzu Scientific Instruments, Columbia, MD, USA) at 380 nm excitation and 460 nm emission wavelengths. Fluorometric calibration was performed with 7-amino-4-methyl-coumarin (AMC) as a reference standard, in the same reaction buffer used for the fluorogenic peptidyl substrates. The resulting AMC calibration curve showed a strong correlation coefficient (r = 0.9987).

### 2.4. Effect of K11777 on the T. evansi CPs In Vitro

Parasite lysates (100 µg each) were pre-incubated with K11777 (0, 1, 10, 100 and 1000 nM in 10% DMSO) for 5 min at RT in 100 µL 50 mM sodium acetate buffer with 10 mM DTT, pH 5.5. The K11777 vinyl sulfone was custom synthesized by Seres Laboratories Inc. (Santa Rosa, CA, USA). Assays were carried out in duplicate and initiated by addition of an equal volume of 40 µM Z-Phe-Arg-AMC in the same buffer. Fluorescence was measured for 5 min at RT in a Multiskan fluorimeter (Thermo Fisher Scientific, Walthham, MA, USA) (380/460 excitation/emission wavelengths) and the data analyzed by non-linear regression (curve fit) of dose–response inhibition, using GraphPad Prism version 11.0.0 for Windows, (GraphPad Software, Boston, MA, USA, www.graphpad.com).

### 2.5. Radiolabeling of the T. evansi CPs

Parasite lysates were prepared by sonication in 50 mM sodium acetate buffer, pH 6.8, followed by clarification and re-extraction, as previously described [51,52]. To assess radiolabeling specificity, the combined parasite supernatants (100 µg per assay) were preincubated with 10% DMSO or 10 µM of the unlabeled CP inhibitors, benzyloxycarbonyl-phenylalanyl-alanine diazomethane (Z-Phe-Ala-CHN_2_) (Bachem, Torrance, CA, USA) or K11777, for 30 min at RT. After the addition of iodinated morpholinourea-leucinyl-homophenylalanine vinyl sulfone phenol (^125^I-LHVS-PhOH; 10^6^ cpm per sample) in 100 µL 50 mM Tris, 5 mM MgCl_2_, 2 mM DTT, pH 5.5, [51,52], the mixture was incubated on ice for 2 h. The reaction was terminated by addition of 4 × SDS sample buffer and analyzed by 12.5% SDS-PAGE [53], followed by autoradiography. The autoradiogram image was saved as a bmp file (350 × 1260 pixels) and analyzed with the Image J software (National Institutes of Health, Bethesda, MD, USA; nih.gov bundled with 64-bit Java Image J 8 (https://imagej.net/ij/download.html, accessed on 21 February 2024).

### 2.6. Purification of the T. evansi Cathepsin L by FPLC and N-Terminal Sequencing

A *T. evansi* lysate (2 × 10^9^ trypanosomes per mL) was prepared in 50 mM sodium acetate buffer, pH 6.8, as previously described. The parasite pellet was reextracted with the same buffer and the supernatants were combined and applied to a MonoQ HR5/5 column (Amersham; Cytiva, Marlborough, MA, USA) that had been previously equilibrated with 20 mM Tris-HCl, pH 8.0. An acetonitrile gradient (0.04–0.3 M) was applied, and 0.5 mL fractions were collected at a flow rate of 0.5 mL/min. The protein concentration of the fractions was determined using the BCA Reagent (Pierce, Rockford, IL, USA) and hydrolysis of the preferred Z-Phe-Arg-AMC substrate (20 µM) was performed, as previously described for the K11777 in vitro assay. For immunoblotting, aliquots of the starting material, the wash and the collected fractions (5 µg each) were subjected to SDS-PAGE (10% gel) electrophoresis, blotted onto PVDF (Millipore, Burlington, MA, USA) and incubated with rabbit anti-*Tbr*CAT L antiserum at a 1/4000 dilution [51]. The secondary antibody, horseradish peroxidase-conjugated goat anti-rabbit IgG (Santa Cruz Biotechnology, Dallas, TX, USA), was used at a 1/5000 dilution, and the blot was developed with the ECL reagent (Amersham; Cytiva, Marlborough, MA, USA).

To sequence the amino-terminal of the CATL peptidase, an aliquot of MonoQ column fraction #10 (200 µL) containing the p40 polypeptide band was transferred to a PVDF membrane (Immobilon-P Millipore) at 23 V for 50 min and sequenced by Edman degradation (UCSF Biomolecular Resource Center). The resulting pentapeptide sequence was analyzed by blastp using the https://blast.ncbi.nlm.nih.gov/Blast.cgi (accessed on 29 July 2026) server [54], against the UniProtKB/Swiss-Prot database. Search parameters were adjusted for a short input sequence, and the search was limited to Trypanosomatidae (taxid:5654) records.

### 2.7. Effect of K11777 on Parasite Survival In Vivo

The experimental *T. evansi* mice infections were performed with three-week-old, female BALB/c mice that were intraperitoneally (IP) injected with the Venezuelan TeAp-ElFrío strain (5 × 10^4^ parasites) [45,55,56]. Controls for these experiments were *T. evansi-*infected mice that received either no treatment (*n* = 6) or 100 µL 10% DMSO in PBS (*n* = 5), also at 12 hpi and then twice daily, as described below for the two K11777 experiments.

The dosing regimens used in the in vivo experiments with K11777 were based on previous assays using a mouse model of *T. cruzi* infection [57]. Parasitemia was estimated every 2–4 days by wet blood film examination, using the “matching method” described by Herbert and Lumsden [58]. This method provides a rapid microscopic estimate of patent parasitemia in the blood, i.e., above antilog 5.4 (250,000) parasites/mL. Mouse survival and signs of disease (e.g., lethargy, lack of social behavior and trans-grooming) were recorded daily for 80 days, at which time the surviving animals were euthanized.

For K11777 Experiment 1, mice (*n* = 5) received 100 µL K11777 (50 mg/mL in 10% DMSO) intra-peritoneally (IP) 12 hpi and twice daily for the following 14 days. Four out of these five K11777-treated mice received additional doses (50 mg/mL IP) on days 16, 18 and 20 dpi (Group 1) or 30 and 34 dpi (Group 2).

For K11777 Experiment 2, mice (*n* = 3) were treated with the same dose regimen of inhibitor, 100 µL of 50 mg/mL IP, beginning 12 hpi, and then twice daily for 17 consecutive days. Survival and signs of disease, (e.g., lethargy, lack of social behavior and trans-grooming) were monitored daily for 52 days, at which point the surviving animals were euthanized. Animals that had parasitemia between 5 × 10^8^ and 2 × 10^9^ parasites/mL or those that displayed signs of disease, (e.g., lethargy, lack of social behavior and trans-grooming) were euthanized. Data from the survival curves were analyzed with Logrank (Mantel–Cox test) using the GraphPad Prism version 11.0.0 for Windows: (GraphPad Software, Boston, MA, USA, www.graphpad.com).

## 3. Results

### 3.1. Identification of CPs in T. evansi Lysates

**Figure 1:**
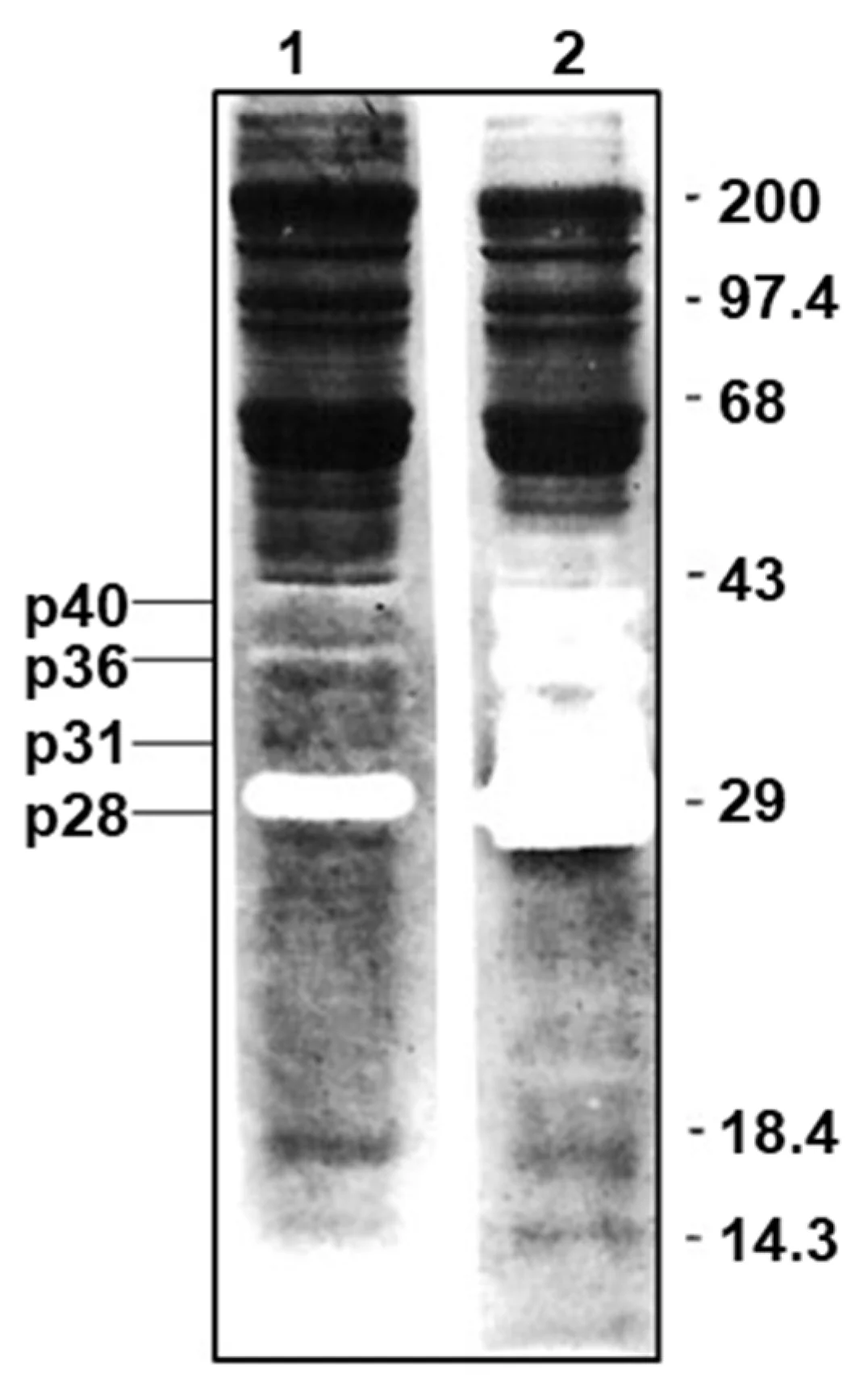
Activation of *T. evansi* peptidases by cysteine. *T. evansi* lysates (20 μg/lane) were electrophoresed through 12.5% polyacrylamide gels containing 0.1% gelatin. After SDS-PAGE, peptidase activation was carried out in 100 mM Tris-HCl buffer, 2.5 mM CaCl_2,_ pH 7.2, at 37 °C for 30 min, in the absence (lane 1) or in the presence (lane 2) of 25 mM cysteine. Peptidase bands were revealed by staining with 3% Coomassie Brilliant Blue R-250 in water: methanol: acetic acid (50:40:10) followed by destaining in the same solution without dye until the appearance of clear bands. Molecular weight markers (GIBCO/BRL) are indicated on the right.

**Figure 2:**
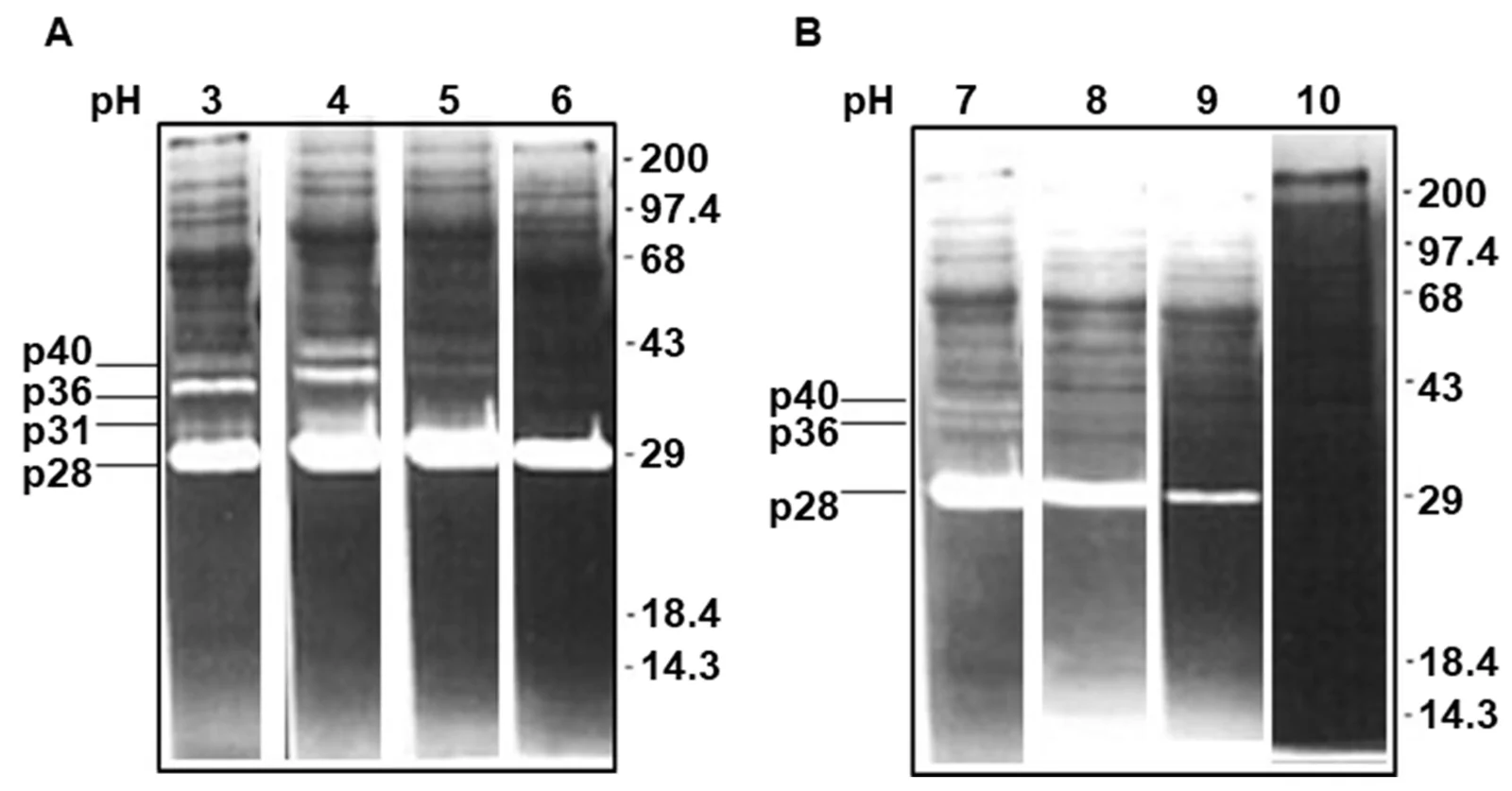
Effect of pH on *T. evansi* peptidase activity. *T. evansi* lysates (20 μg/lane) were electrophoresed through 12.5% polyacrylamide gels containing 0.1% gelatin. Activation was performed in 100 mM sodium citrate buffer, pH 3.0–6.0 (panel (**A**)), or 100 mM Tris-HCl, pH 7.0–8.0, or 100 mM sodium bicarbonate, pH 9.0–10.0 (panel (**B**)), in the presence of 25 mM cysteine at 37 °C for 30 min. Peptidase bands were revealed by staining with 3% Coomassie brilliant blue R-250 in water: methanol: acetic acid (50:40:10) followed by destaining in the same solution without dye until the appearance of clear bands. Molecular weight markers (GIBCO/BRL, Bethesda, MD, USA) are indicated on the right.

In the presence of 25 mM cysteine, four peptidase bands, with relative mobilities of 28, 31, 36 and 40 kDa (p28, p31, p36 and p40), were identified in soluble *T. evansi* lysates using gelatin zymography at neutral pH (Figure 1, lane 2). In the absence of cysteine, only the p40, p36 and p28 bands were seen, all with decreased intensity (Figure 1, lane 1), suggesting that they correspond to CPs. The pH-dependence of these peptidases was analyzed in four buffers ranging from pH 3.0 to 10.0 (Figure 2). The p28 peptidase was active across a broad pH range, between 3 and 9, with maximal degradation at pH 4 and 5 (Figure 2A). The p31, p36 and p40 gelatinase activities were detected in the pH 3−5 interval (optimal between pH 3.0 and 4.0) and at pH 7.0. A significant inhibition of all the CPs was observed between pH 8.0 and 10.0 (Figure 2B).

**Figure 3:**
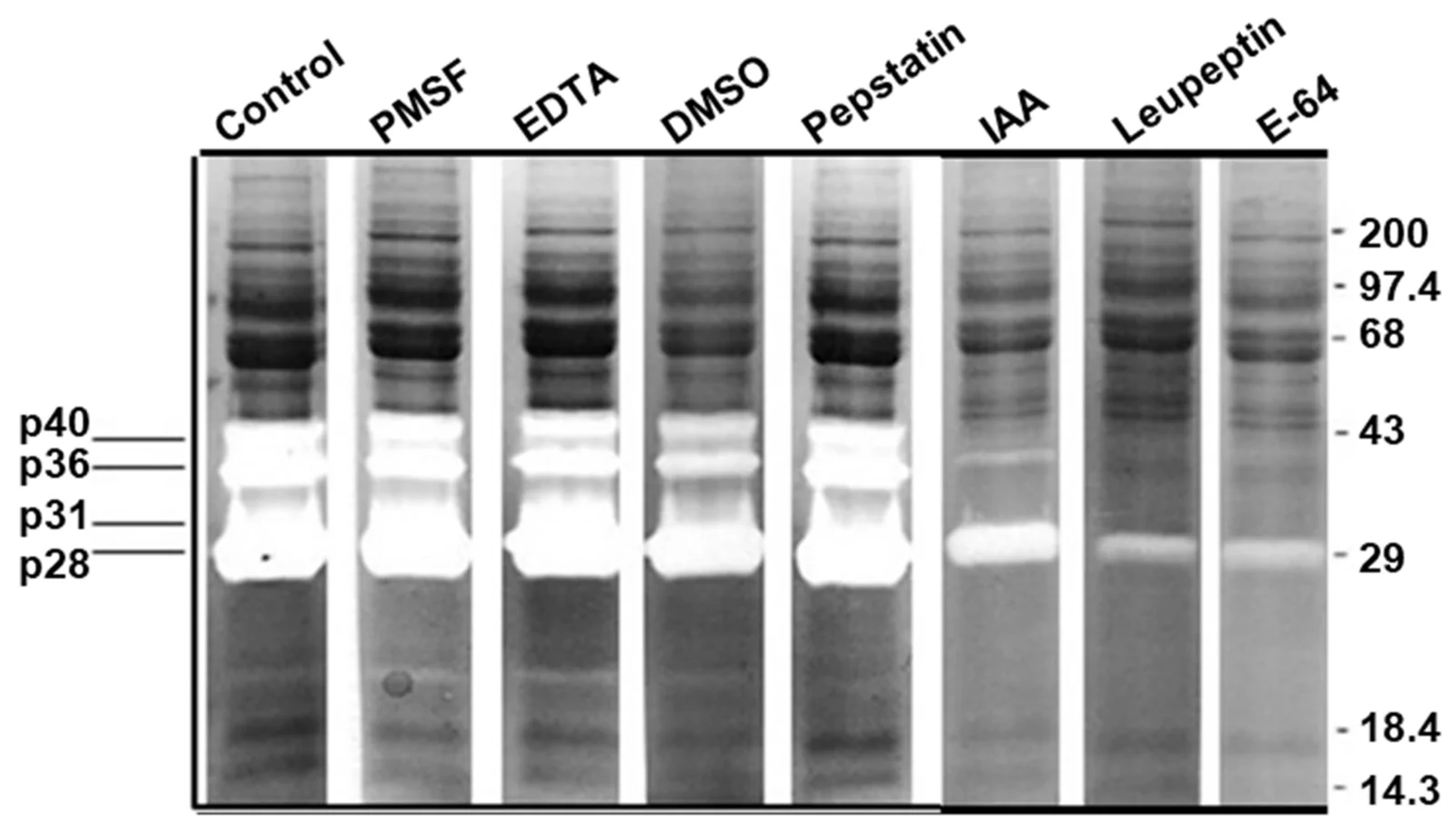
Effect of protease class-specific inhibitors on *T. evansi* peptidases. *T. evansi* lysates (20 μg/lane) were electrophoresed through 12.5% polyacrylamide gels containing 0.1% gelatin. Gels were incubated in 100 mM sodium citrate, 25 mM cysteine, pH 4.0, for 30 min at 37 °C with 1 mM PMSF, 10 mM EDTA, 1 μM pepstatin,1 mM IAA, 20 μM leupeptin, 10 μM E-64 or 10% DMSO as an inhibitor control. Peptidase bands were revealed by staining with 3% Coomassie Brilliant Blue R-250 in water: methanol: acetic acid (50:40:10) followed by destaining in the same solution without dye until the appearance of clear bands. Molecular weight markers (GIBCO/BRL, Bethesda, MD, USA) are indicated on the right.

Various peptidase class-specific inhibitors were used to further characterize the enzymatic activities in the *T. evansi* lysate (Figure 3). Four bands, p28, p31, p36 and p40, were observed in the control assays with no inhibitors or with the 10% DMSO solvent (Figure 3). The peptidases were strongly inhibited by the two CP inhibitors, IAA and E-64, and by leupeptin, which targets both CPs and serine peptidases (Figure 3). In contrast, three inhibitors had no effect: PMSF, which inhibits serine peptidases; EDTA, a metallo-peptidase inhibitor; and pepstatin, which is selective for aspartyl peptidases (Figure 3). Based on their activation by cysteine, patterns of inhibition in the presence of CP inhibitors, as well as their pH profiles, the four *T. evansi* bands with peptidase activities were designated as CPs.

### 3.2. Degradation of Equine Proteins and Fluorogenic Peptides by T. evansi Peptidases

**Figure 4:**
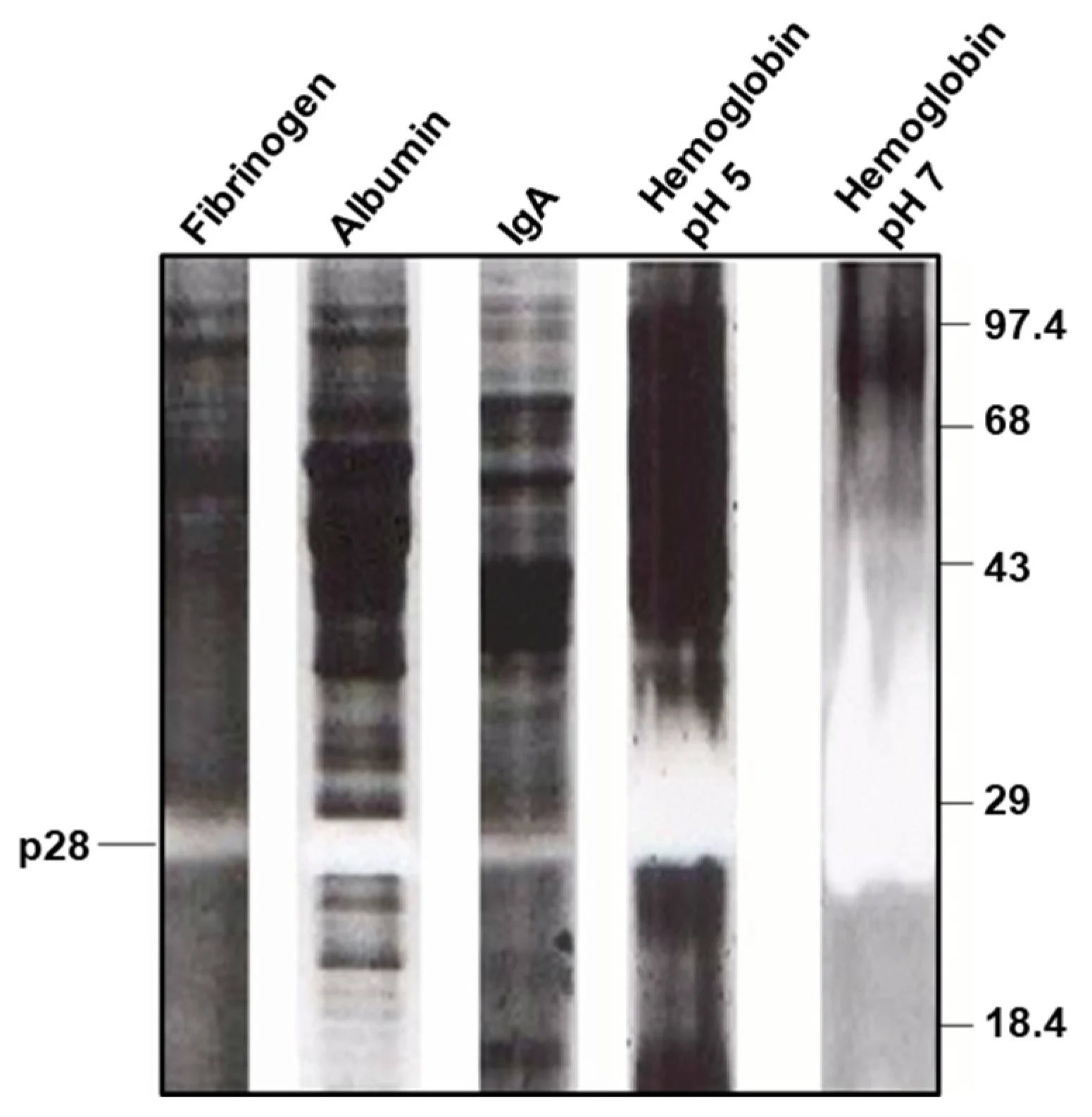
Degradation of equine proteins by *T. evansi* lysate. Equine fibrinogen, albumin and IgA (0.03%) were conjugated in 12.5% polyacrylamide gels and evaluated as substrates for *T. evansi* lysates (20 μg/lane). Zymograms were activated for 30 min at 37 °C in 100 mM Tris-HCl, 25 mM cysteine, 2.5 mM CaCl_2_, pH 7.0, whereas hemoglobin was tested under two different pH conditions: 100 mM sodium citrate, pH 5.0, and 100 mM Tris-HCl buffer, pH 7.0, containing the same final concentrations of cysteine and CaCl_2_. Peptidase bands were revealed by staining with 3% Coomassie Brilliant Blue R-250 in water: methanol: acetic acid (50:40:10) followed by destaining in the same solution without dye until the appearance of clear bands. Molecular weight markers are indicated on the right.

Because *T. evansi* is a hemotropic parasite and equids are one of its preferred hosts, we evaluated the ability of the parasite CPs to degrade the major equine proteins, hemoglobin, fibrinogen, albumin, and IgA, the dominant immunoglobulin isotype present in mucosal membranes, body secretions and blood [59]. One of the previously identified CPs, p28, degraded fibrinogen, albumin, and IgA in zymograms at pH 7.0 (Figure 4), and showed strong hemoglobinolytic activity at pH 7.0 and 5.0 (Figure 4).

**Figure 5:**
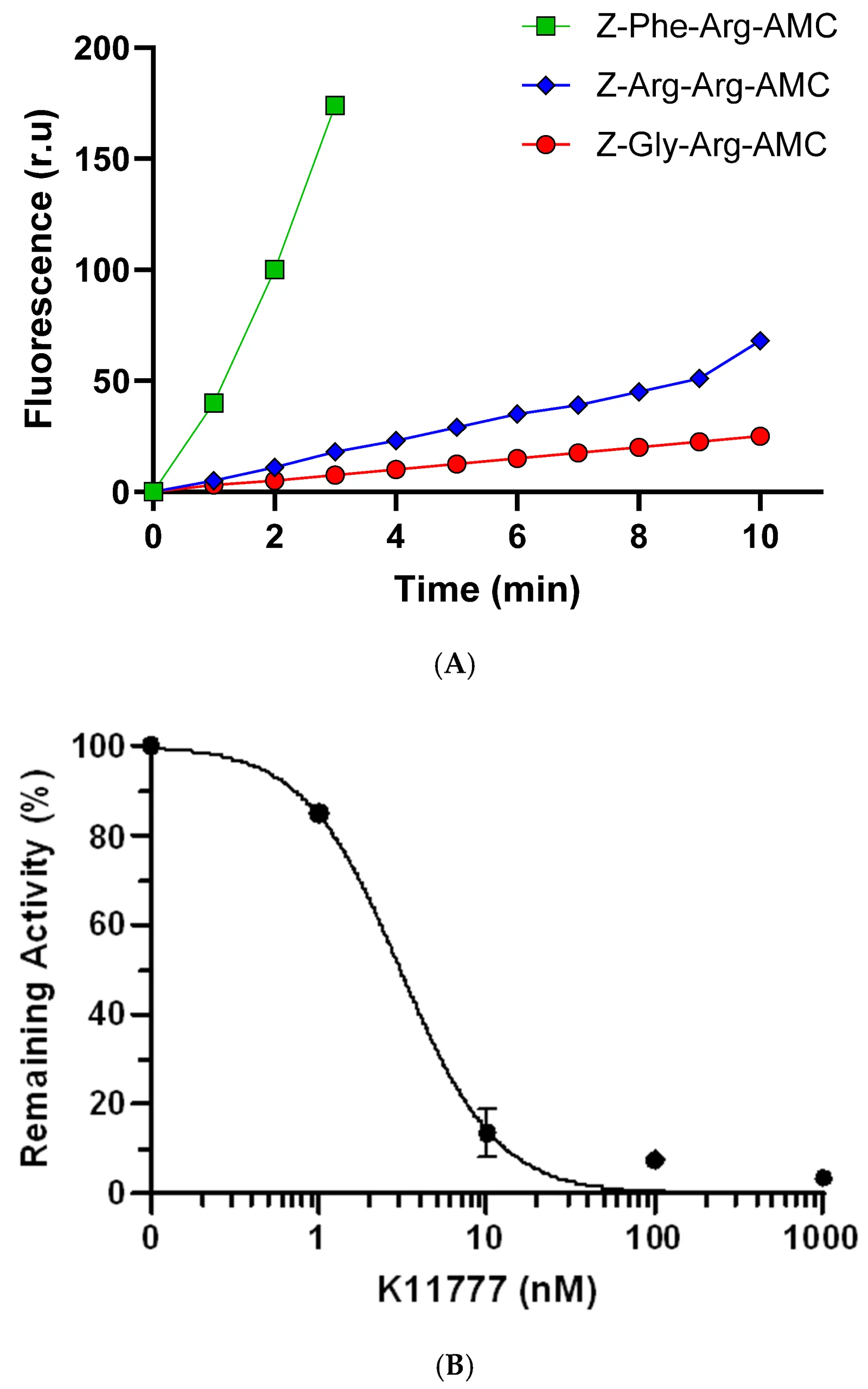
(**A**) Hydrolysis of fluorogenic peptidyl substrates by *T. evansi* lysate. Lysate from 2 × 10^6^ trypanosomes/mL was tested with 4 μM Z-Phe-Arg-AMC, Z-Arg-Arg-AMC or Z-Gly-Arg-AMC in 2 mL 100 mM sodium phosphate, pH 7.0, containing 10% DMSO and 2 mM DTT, at 30 °C. Fluorescence was measured every 60 s for 10 min in a Shimadzu Plus fluorimeter (λ_exc_ = 380 nm; λ_emission_ = 460 nm). (**B**) Inhibition of *T. evansi* CP activity by K11777. CP assays (duplicates) were initiated by pre-incubating parasite lysate (100 µg protein) with K11777 at 0, 1, 10, 100 and 1000 nM in 10% DMSO for 5 min at RT in 0.1 mL 50 mM sodium acetate, 10 mM DTT, pH 5.5. Z-Phe-Arg-AMC (20 µM in 0.1 mL) was added and incubated for 5 min at RT. Fluorescence was measured in a Multiskan fluorimeter (λ_exc_ = 380 nm; λ_emission_ = 460 nm). An apparent IC_50_ = 3.06 nM (95% CI: 2.15–4.47 nM, Rsquare = 0.9893) was calculated using a non-linear regression (best fit values) log_10_ inhibitor vs. normalized response-variable slope with the GraphPad Prism 11.02 statistics software.

The CP activities present in the *T. evansi* lysate were further examined in solution assays with three peptidyl-AMC substrates (Figure 5A). Z-Phe-Arg-AMC was the preferred substrate with 7× and 17× higher hydrolysis rates relative to Z-Arg-Arg-AMC and Z-Gly-Arg-AMC, respectively (Figure 5A). The cleavage of Z-Phe-Arg-AMC by the *T. evansi* lysate was completely inhibited by K11777 in a concentration-dependent fashion with an apparent IC_50_ of 3.06 nM (95% CI: 2.15–4.47 nM, R_squared_ = 0.9893) (Figure 5B). These results indicate that the CPs in the *T. evansi* lysate belong to the papain-superfamily and can be classified as cathepsin-like enzymes.

### 3.3. Active Site Radiolabeling Identifies Major CPs in T. evansi Lysates

**Figure 6:**
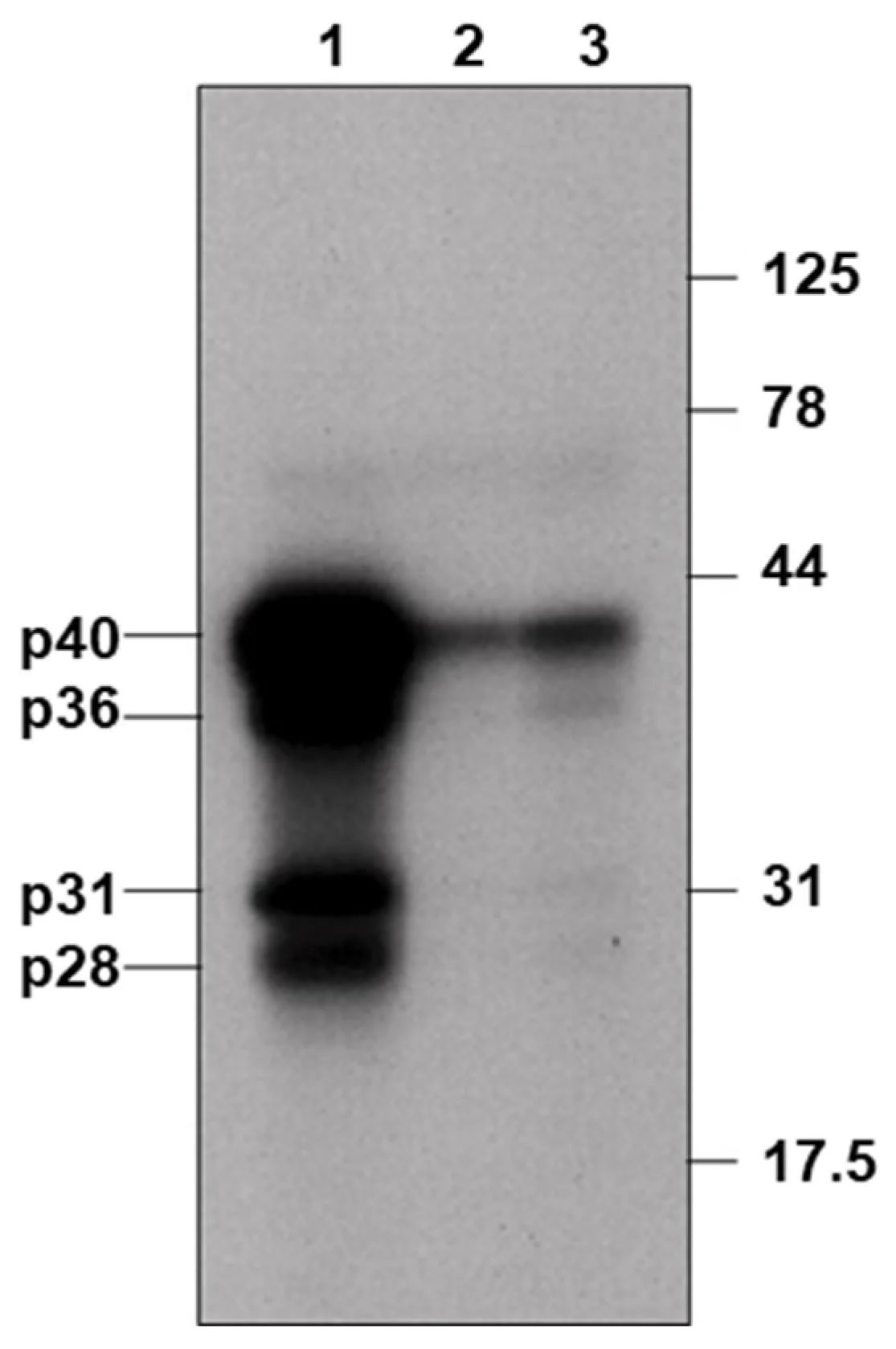
Active site radiolabeling of *T. evansi* extracts. Parasite lysate (100 μg) was incubated with the radiolabeled active-site probe, ^125^I-LHVS- PhOH, on ice for 2 h, followed by SDS-PAGE (12.5%) and autoradiography. To evaluate the specificity of binding by ^125^I-LHVS- PhOH, lysates were preincubated for 30 min at RT with 10% DMSO (control, lane 1), or 10 µM Z-Phe-Ala-CHN_2_ (lane 2) or K11777 (lane 3), prior to radiolabeling. The image was analyzed with the NIH Image J software bundled with 64-bit Java Image J 8 (https://imagej.net/ij/download.html, accessed on 21 February 2024). Molecular weight markers (GIBCO/BRL) are indicated on the right.

The active-site-directed peptidyl inhibitor ^125^I-LHVS-PhOH identified CPs with relative mobilities of 28, 31, 36 and 40 kDa (Figure 6, lane 1). Densitometric analysis showed that binding of ^125^I-LHVS-PhOH to these CPs was reduced by approximately 80%, after a 30 min preincubation with 10 µM of either of two CP inhibitors, Z-Phe-Ala-CHN_2_ (lane 2) or K11777 (lane 3).

### 3.4. N-Terminal Sequencing Confirms the Identity of the Cathepsin L Peptidase

**Figure 7:**
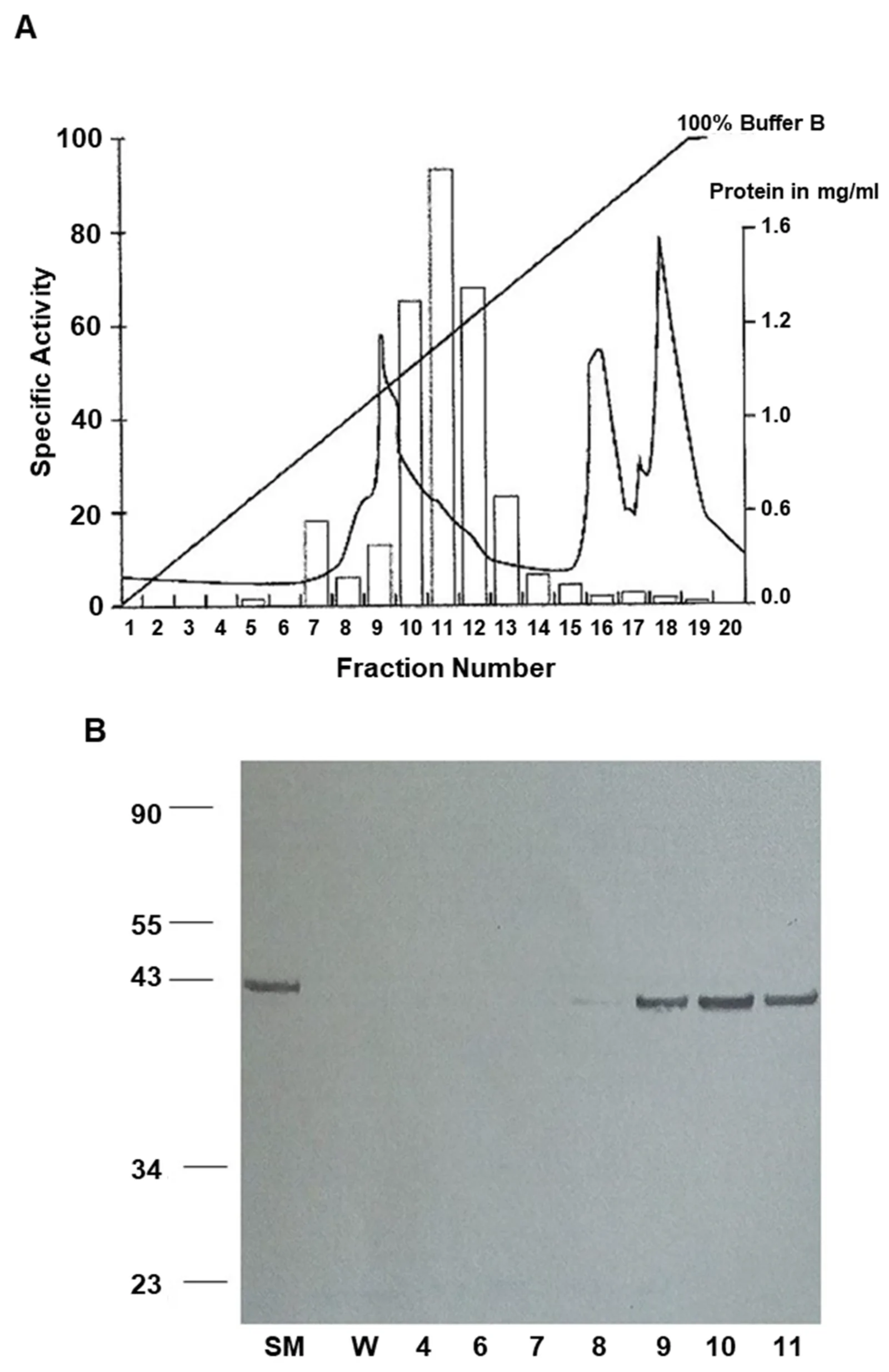
Purification of the *TevCATL* by Mono Q-FPLC Chromatography and Western blotting with anti-*Tbr*CATL antibodies. (**A**) *T. evansi* lysate (2 × 10^9^ trypanosomes/mL) was separated using a MonoQ HR5/5 column equilibrated with 20 mM Tris-HCl, pH 8.0, and a 0.04–0.3 M acetonitrile gradient, as indicated (straight line). Fractions (0.5 mL each) were collected at a flow rate of 0.5 mL/min, quantitated by the BCA reagent (---) and tested for CATL activity with the Z-Phe-Arg-AMC substrate (20 μM) in 50 mM sodium acetate, 10 mM DTT, pH 5.5 (bars). (**B**) Aliquots of the starting material (SM), wash (W, fractions 1–3) and fractions 4–11 (5 µg) were subjected to 10% SDS-PAGE, blotted onto PVDF, and reacted with monospecific rabbit antibodies raised to the recombinant *Tbr*CATL (1/4000 dilution). Horseradish peroxidase-conjugated goat anti-rabbit IgG was employed as the secondary antibody (1/5000 dilution), and the blot was developed using the ECL detection reagent (Amersham, Cytiva, MA, USA).

The 40 kDa *T. evansi* CP was purified in a single chromatographic step, using MonoQ FPLC and an acetonitrile gradient (Figure 7). Figure 7A shows that the cathepsin activity, measured by the hydrolysis of the Z-Phe-Arg-AMC (fractions 9–13), was eluted at the tail of the first broad protein peak. Analysis by SDS-PAGE and immunoblotting with rabbit monospecific antibodies to the recombinant *Tbr*CATL revealed the 40 kDa cross-reacting species in the starting material and in fractions 8–11 (Figure 7B). Partial Edman sequencing of the 40 kDa cathepsin polypeptide in fraction 10 returned an N-terminal pentapeptide sequence: APAAV. The APAAV pentapeptide has been recognized as an autoproteolytic cleavage signal and the N-terminal sequence of Cruzipain, the orthologous cathepsin L peptidase from *T. cruzi*, following removal of the proregion [60,61]. This motif has also been found in other papain-like cysteine peptidases. Our PSI-BLAST search of the UniProtKB/SwissProt database [54,62] showed that the pentapeptide sequence APAAV is identical, with a perfect match (5/5) to the N-terminus of the mature CATL peptidases from *Trypanosoma cruzi* and *T. b. brucei* [60,61,63] confirming the p40 species as *Tev*CATL.

### 3.5. K11777 Is Parasiticidal In Vivo and Extends the Survival of Mice Infected with T. evansi

**Figure 8:**
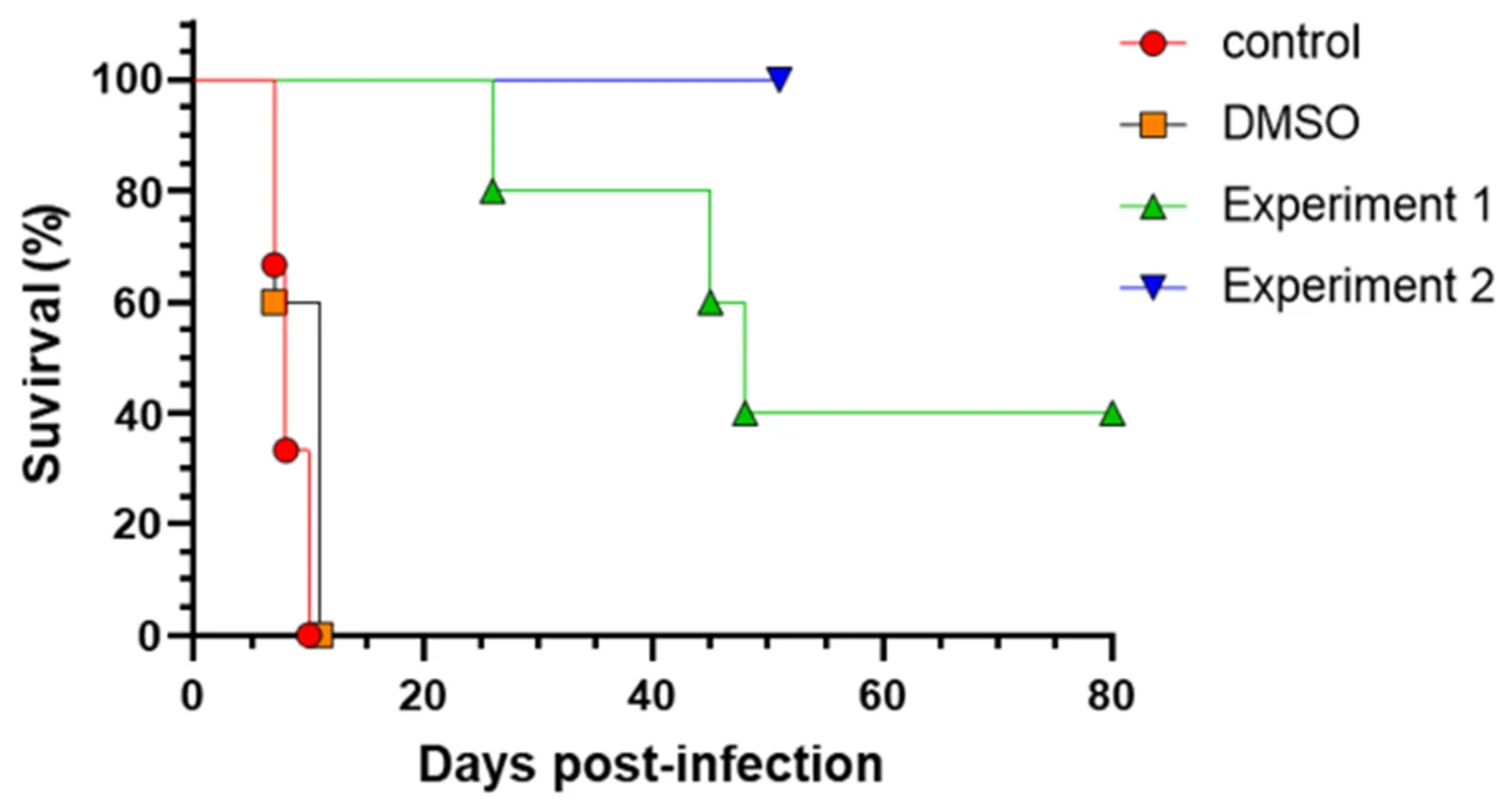
Effect of K11777 on the survival of mice infected with *T. evansi.* Three-week-old, female BALB/c mice were infected IP with 5 × 10^4^ parasites. Control groups either received no treatment (*n* = 6) or vehicle 10% DMSO (*n* = 5). In Experiment 1, mice (*n* = 5) received 100 µL of K11777 (50 mg/mL, IP in 10% DMSO) 12 hpi and twice daily for the subsequent 14 days. Four of the five treated mice received equal doses on days 16, 18 and 20 pi (Group 1: # 2 and #3), or on days 30 and 34 pi (Group 2: # 4 and #5). In Experiment 2, mice (*n* = 3) were treated with 100 µL of K11777 (50 mg/mL, IP in 10% DMSO), beginning 12 hpi, and then twice daily for 17 days. Mice survival was monitored for 80 days (Expt. 1) or for 52 days (Expt. 2), at which time the surviving animals were euthanized. Animals with parasitemia between 5 × 10^8^–2 × 10^9^ parasites per mL or displaying signs of severe disease, including lethargy or lack of social behavior and trans-grooming, were euthanized. Survival analyses (Kaplan–Meier) using GraphPad Prism 11.02 showed statistically significant differences between the control, the DMSO control and the K11777-treated infected mice with *p* values < 0.0001 (Logrank test for trend).

**Figure 9:**
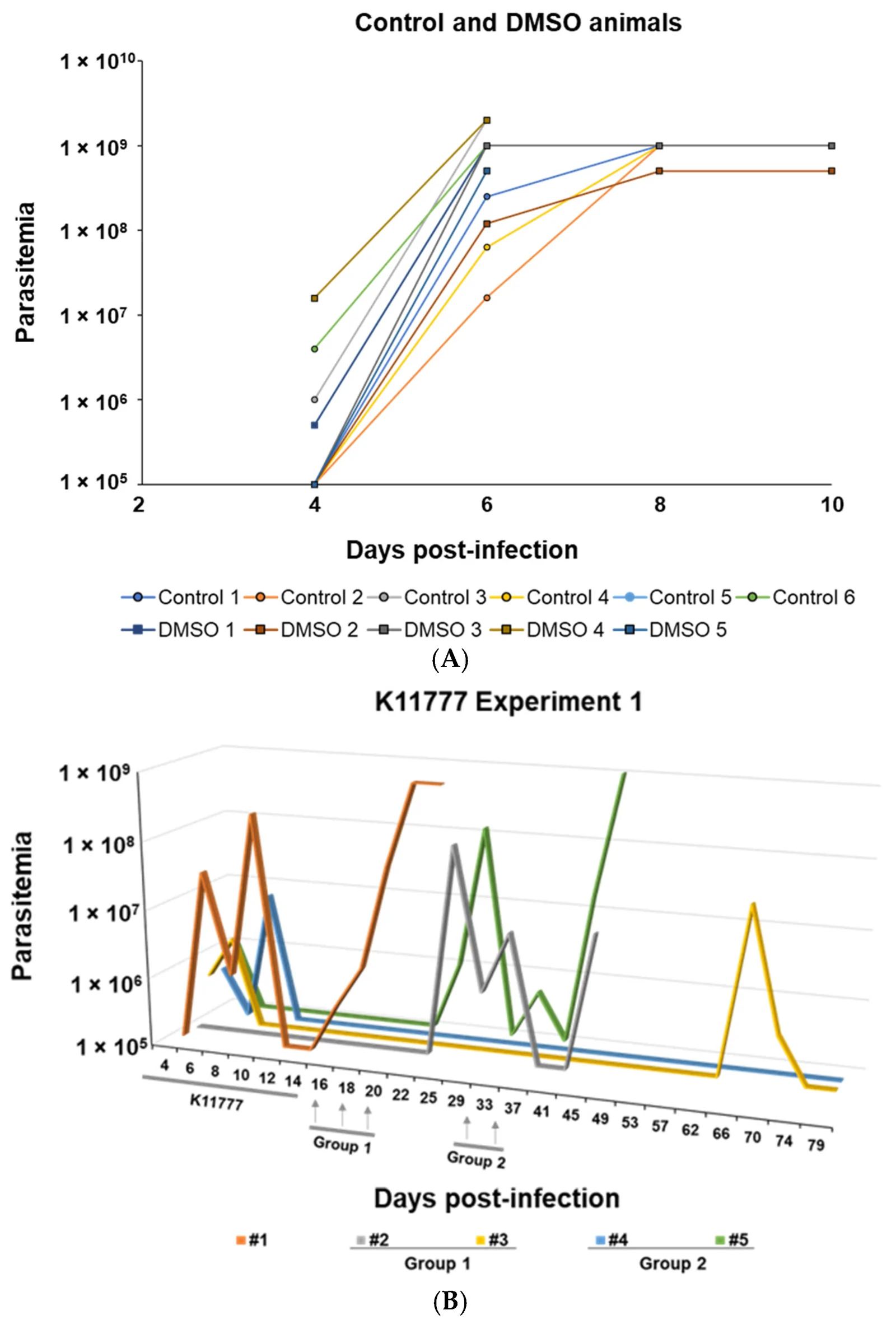

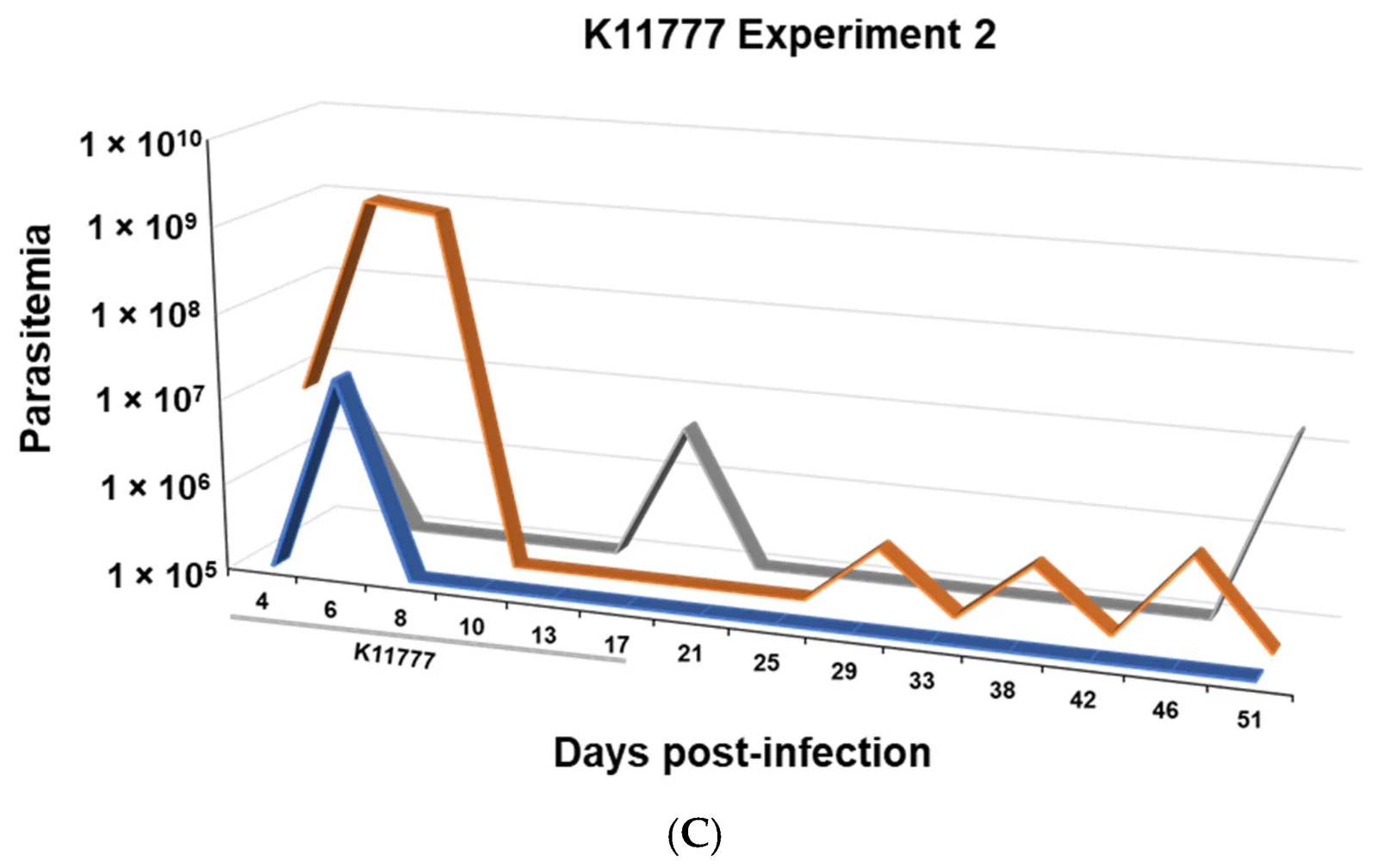
Effect of the K11777 on blood parasitemia and survival of *T. evansi*-infected mice. Three-week-old, female BALB/c mice were infected IP with 5 × 10^4^ parasites. Parasitemia was estimated every 2 days by wet blood film examination, using the Herbert and Lumsden “matching method” [58]. Animals with parasitemia reaching between 5 × 10^8^ and 2 × 10^9^ parasites/mL and/or displaying lethargy, or lack of social behavior and trans-grooming, were euthanized. (**A**) Non-treated and DMSO vehicle control experiments. The control *T. evansi*-infected mice received no treatment (*n* = 6) and the vehicle control mice (*n* = 5) received 100 µL 10% DMSO starting 12 hpi and twice daily through 10 dpi. (**B**) In K11777 Experiment 1, the *T. evansi*-infected mice (*n* = 5) received a single IP dose of 100 µL K11777 (50 mg/mL in 10% DMSO) administered 12 h pi and then twice daily for the next 14 days. Two of the five mice then received additional 50 mg/mL IP doses on days 16, 18 and 20 pi (Group 1), whereas two other mice received additional K11777 IP (100 μL of 50 mg/mL) doses on 30 and 34 dpi (Group 2), as indicated by the arrows. (**C**) In K11777 Experiment 2, the *T. evansi*-infected mice (*n* = 3) were treated with a single IP dose of 100 µL K11777 (50 mg/mL in 10% DMSO) 12 hpi and then twice daily for the next 17 days.

We then examined the effect of the CP inhibitor K11777 on the course of parasitemia and survival of *T. evansi*-infected BALB/c mice, compared to control mice that received no treatment or vehicle (10% DMSO) alone (Figure 8 and Figure 9). Vehicle control mice were euthanized 7–10 dpi (Figure 8) after showing signs of lethargy, non-social behavior and/or parasitemia values between 5 × 10^8^ and 2 × 10^9^ parasites/mL (Figure 9A). The parasitemia of the non-treated mice reached maximum values from 5 × 10^8^ to 1 × 10^9^ par/mL between 7 to 10 pi, at which time they were euthanized (Figure 8 and Figure 9A).

In comparison to the 10 dpi survival of the experimental control animals, treatment with K11777 for 14 consecutive days (Figure 8 and Figure 9B, Expt. 1, *n* = 5) with no further treatment (Figure 9B, #1) resulted in a 26 dpi survival. With additional doses of K11777 to Group 1 (#2 and # 3) (Figure 9B), mice survival was extended to 45 and 80 dpi, at which point the experiment was terminated. In Group 2 (#4 and #5) (Figure 9B), that also received additional doses of K11777, mouse #5 survived to 48 dpi, while mouse #4 survived to 80 dpi, at which point Experiment 1 was terminated. Despite the small number of mice in the subgroups, administration of additional doses of K1177 on 16, 18 and 20 dpi (Group 1), or on days 30 and 34 dpi (Group 2), had a beneficial effect on mice survival.

Treatment with K11777 for 17 consecutive days (Expt. 2) (Figure 8 and Figure 9C) also extended mice survival (*n* = 3) from the 10 dpi observed for the control *T. evansi*-infected mice to 52 dpi, at which point Experiment 2 was terminated. Statistical analyses (Kaplan–Meier) and Logrank test (*Mantel–Cox method) demonstrated the significant differences (*p* < 0.0001) in survival between the controls and the K11777-treated mice (Figure 8).

The parasitemia of the non-treated and DMSO-treated mice increased exponentially, reaching maximum values ranging from 5 × 10^8^ to 2 × 10^9^ parasites/mL between days 6 and 10 pi (Figure 9A). In contrast, the K11777-treated mice were able to at least partially control parasite proliferation (Figure 9B,C). In Experiment 1 (Figure 9B), two of the five K11777-treated mice (#3 and #4) survived for the duration of the study (80 dpi), whereas the remaining three mice showed partial parasitemia control during the first three to six wpi, with undulating parasitemia waves, until euthanasia on days 26, 45 and 48 pi, after exhibiting clinical signs of disease, non-social behavior and/or high parasitemia. In Experiment 2 (Figure 9C), parasite proliferation was well controlled, and the three mice survived for the duration of the study, 52 dpi. The twice-daily treatment with K11777, for 17 days, during the initial phase of the infection, resulted in partial parasitemia control and a significant increase in survival. Overall, the administration of the K11777 inhibitor provided a significant therapeutic benefit to *T. evansi*-infected mice.

## 4. Discussion

In this study, we identified and characterized p28, p31, 36 and p40 CP activities in a Venezuelan *T. evansi* strain using gelatin-gel zymography, peptidase class-specific inhibitors and peptidyl substrates. We also demonstrated a clear therapeutic benefit in *T. evansi*-infected mice treated with the CP inhibitor, K11777.

Acidic, thiol-dependent hemoglobinolytic activities have been documented in several kinetoplastid species, including *T. brucei*, *T. evansi*, *T. equiperdum*, *C. fasciculata* and *Leishmania tarentolae* [64]. In keeping with the nomenclature for papain and other thiol-dependent enzymes, the term “trypanopains” was coined to refer to trypanosome CPs [65]. The Venezuelan *T. evansi* CPs share common characteristics with the p42 and p28 trypanopains present in an African *T. evansi* camel isolate, and with CPs found in *T. b. brucei* and *T. b. gambiense* [65], including their size, pH profile and ability to degrade fibrinogen. Similarly, gelatinase activities (29, 32, 35 and 40 kDa) have been reported in *T. evansi* and *T. equiperdum* lysates [66], while a more complex set of eight peptidases (28, 32, 35, 41, 69, 95, 105 and 150 kDa) was described in an Indian *T. evansi* camel isolate [67].

The p31, p36 and p40 *T. evansi* CP activities identified here exhibit a narrow pH optimum of ~4.0, consistent with a lysosomal location. In contrast, the p28 activity showed a much broader activity range, between pH 3.0 and 9.0, and hydrolyzed all four equine protein substrates tested at pH 5.0, as well as hemoglobin at pH 7.0, suggesting a possible role in parasite nutrition and avoidance of host defense mechanisms. Other trypanosome CPs, including the *T. b. brucei* CP [68], the *T. cruzi* CP, cruzain [69] and the recombinant *Tb*rCATL [51,70], have been localized to lysosomes or lysosome-like organelles, where they participate in the degradation of parasite and host proteins. Several peptidases, including the *T. b. gambiense* cathepsins and *Tbr*CATL, are released or secreted into the extracellular milieu and have been linked to signal transduction cascades promoting *T. brucei* passing the blood–brain barrier into the CNS, as well as cardiac dysfunction via perturbation of intracellular calcium levels [31,32,71,72]. Future studies are needed to elucidate the role of *T. evansi* CPs in parasite survival and host–parasite interaction.

The *T. evansi* CPs preferentially hydrolyzed Z-Phe-Arg-AMC relative to Z-Arg-Arg-AMC AMC and Gly-Arg-AMC at neutral pH, and were inhibited by K11777, a potent and irreversible pan-cathepsin inhibitor [20,29,37,38,39,40,41,42,43]. Gly-Arg-AMC is a specific substrate for trypsin-like peptidases [73], while the *T. evansi* CPs preference for a bulky, hydrophobic Phe residue at the substrate P2 position is consistent with previous data for the recombinant cathepsins, *Tbr*CATL and *Tb*CATB [51,74]. Furthermore, the K11777 apparent IC_50_ value of 3.06 nM that we determined, using the crude *T. evansi* lysate, closely mirrors those reported for the purified recombinant orthologous peptidases, Cruzain and Rhodesain (1.5–8 nM) [75].

The *T. evansi* cathepsins, p28, p31, 36 and p40, resolved by gelatin zymography, were also labeled with the ^125^I-LHVS- PhOH active-site probe, which was originally designed to identify mammalian CPs. The same probe previously identified cathepsins with similar relative motilities (29, 33 and 40 KDa) in *T. b. brucei* lysates [51]. In addition, the purified p40 *T. evansi* CP was recognized by the monospecific antibodies developed to recombinant *Tbr*CATL [51], providing further evidence of the close relationship between these two CPs. Edman degradation determined the N-terminal sequence of p40 as APAAV, which is identical to the N-termini of the catalytic domains of other trypanosome C1A orthologs, including the *T. brucei* CP [63], the *T. cruzi*, cruzain [60,61] and the CATL peptidases present in *T. dionisii* and *T. rangeli* [76]. Both the N-terminal sequence of the purified p40 *T. evansi* cathepsin and its cross-reactivity with the anti-*Tbr*CATL antiserum are consistent with its classification as a CATL. Based on the post-translational processing reported for *Tbr*CATL [51,70] and cruzain [77], we propose that p40 corresponds to the mature glycosylated peptidase, after autoproteolysis of its N-terminus, but bound to its 11.1 kDa C-terminal domain, whereas p28, p31 and p36 are active isoforms generated by further self-proteolysis and glycosylation. In keeping with suggested nomenclature [19,78], we use the term *Tev*CATL to describe p40 and its differentially processed p28, p31 and p36 products.

Kinetoplastid CPs contribute to nutrient-acquisition and are involved with signaling pathways that subvert host defense mechanisms to cause pathology and disease [19,20,21,72,79]. Our study shows, for the first time, that the pan-cathepsin irreversible inhibitor, K11777, also known as SLV213, targets the *Tev*CATL both in vitro and in vivo, and extends host survival in a mouse infection model in treated mice. Our results suggest that extending the K11777 treatment from 14 to 17 days leads to a more favorable control of parasite proliferation by the host, as evidenced by the undulating, sub-lethal levels of parasitemia and increased survival. The initial phase of parasite infection in this mouse model appears to be a crucial “window of opportunity” for inhibitor action and activation of host defense mechanisms that ultimately determine parasite multiplication and host survival.

These results underscore the importance of identifying *Tev*CATL as a potential drug target for *T. evansi*. Surra affects domestic and wild animals and *T. evansi,* its causative agent, is the most widely distributed animal trypanosome through Latin America and Northern Africa, above the tsetse belt, the Middle East and Asia [3,4]. Previous reports have shown that Trypanosomatid CPs are essential for infectivity, parasite survival in the host, and have been implicated in pathogenicity [19,20,21,22]. Our findings extend promising drug discovery reports of K11777, which is considered to be a broad spectrum anti-trypanosomal agent, for the treatment of Chagas disease caused by *Trypanosoma cruzi*, a human and zoonotic pathogen [61], and HAT, caused by *T. b. rhodesiense* and *T. brucei gambiense*. In line with the One Health vision [80], the identification of novel and effective therapeutic targets against surra could lead to the development of new drugs to treat or cure other animal trypanosomosis, including nagana, caused by *T. vivax*, *T. congolense*, *T. brucei brucei*, and *T. simiae*, and dourine, a sexually transmitted disease in Equidae, caused by *T. equiperdum*.

The inhibition of TevCATL in vivo, following administration of K11777, resulted in parasite attenuation, perhaps due to a reduced ability of the parasite to evade the host immune response. The orthologous *Tbr*CATL plays a crucial role in virulence and pathogenicity, facilitating parasite entry and trans-endothelial passage through the blood–brain barrier [30,31,79,81]. Our in vivo efficacy data are consistent with those reported by Scory et al. [35] showing that another irreversible CP inhibitor, Z-Phe-Ala-CHN_2,_ effectively decreased parasitemia and prolonged host survival in a *T. b. brucei* mouse model. Other studies, both in vitro and in vivo, have amply demonstrated the trypanocidal activity of small molecule CP inhibitors [36,37,38,39,69,82]. Steverding et al. [33,34] showed that inhibition of the *Tb*CATL alone is sufficient to kill the bloodstream form of *T. brucei*. They demonstrated that the trypanocidal effect of the peptidyl vinyl sulfone LU-102 is due to its selective inhibition of *Tb*CATL over *Tb*CATB, thus confirming *Tb*CATL as an essential drug target.

Significant progress in the treatment of HAT was achieved in 2018 with the approval by the European Medicines Agency (EMA) of fexinidazole, a DNA synthesis inhibitor developed by the Drugs for Neglected Diseases *initiative* (DND*i*) in collaboration with Sanofi [83,84]. Fexinidazole is the first all-oral treatment for the hemo-lymphatic and meningo-encephalitic stages of HAT [85,86]. Due to the high levels of toxicity, undesirable side effects of the current drugs, and the ability of the parasite to develop drug (cross)resistance, the development of novel drugs for animal trypanosomosis and HAT remains a high priority [29,42,43,82,87]. Our results open new opportunities for drug discovery to treat animal trypanosomosis caused by *T. evansi* and other related trypanosomes.

## 5. Conclusions

Animal trypanosomoses, caused by *Trypanosoma brucei brucei*, *T. evansi* and *T. equiperdum*, are widely distributed throughout the globe and are responsible for substantial direct and indirect costs associated with animal protein production. *Trypanosoma evansi*, the most widespread animal trypanosome of African origin, causes the disease known as “surra” [2,3]. In this work, we presented the molecular characterization of four CPs present in a Venezuelan strain of *T. evansi*: p28, p31, p36 and p40. Enzymatic assays with fluorogenic peptidyl substrates and active-site labeling with the ^125^I-LHVS-PhOH vinyl sulfone inhibitor showed that the *T. evansi* CPs belong to the Cathepsin L peptidase C1 family, thus their denomination as *Tev*CATL. The irreversible pan-cathepsin vinyl sulfone inhibitor, K11777, targets the *Tev*CATL both in vitro and in vivo, and it extended host survival in a mouse infection model. The potential of *Tev*CATL as therapeutic target for the treatment of surra extends the promising drug discovery studies with several CP inhibitors against Chagas disease due to *Trypanosoma cruzi*, a human and zoonotic pathogen and HTA, caused by *T. b. rhodesiense* and *T*. *brucei gambiense* and transmitted by the tsetse in sub-Saharan Africa [20,29,33,34,37,38,39,40,41,42,43]. Consequently, and considering the One Health perspective [80], the exploitation of novel drug targets has potential for the development of effective treatments against various pathogenic trypanosomes that affect both humans and animals.

## Data Availability

The raw data supporting the conclusions of this article will be made available by the authors on request.

## Abbreviations

The following abbreviations are used in this manuscript:

CPs	Cysteine peptidases: also known as cysteine proteases, these are proteolytic enzymes that contain a cysteine residue in their catalytic triad.
CATL	Cathepsin L: the name cathepsin originates from the Greek word kathepsein, which means to digest. Cathepsins L and B belong to the Clan CA, C1 family of papain-like cysteine peptidases.
CATB	Cathepsin B: it has dual endo- and exopeptidase activity and plays an essential role in endocytic nutrient processing and parasite survival.
IAA	Iodoacetic acid: an irreversible alkylating inhibitor that binds covalently to the active-site cysteine residue of peptidases.
K11777 (SLV13)	N-methyl-piperazine-phenyl-alanyl-homo-phenylalanyl-vinyl sulfone-phenyl: a potent, broad-range irreversible cysteine protease inhibitor.
PMSF	Phenylmethyl sulfonyl fluoride: an irreversible inhibitor of serine proteases and some cysteine peptidases.
NTDs	Neglected Tropical Diseases: include a diverse group of conditions caused by various pathogens, including viruses, bacteria, parasites, fungi and toxins that are associated with devastating health, social and economic consequences.
HAT	Human African Trypanosomiasis: an NTD caused by *Trypanosoma brucei gambiense* and *Trypanosoma brucei rhodesiense* and transmitted through infected tsetse flies in sub-Saharan Africa.
